# Association between handgrip strength and depression among Chinese older adults: a cross-sectional study from the China Health and Retirement Longitudinal Study

**DOI:** 10.1186/s12877-023-04034-6

**Published:** 2023-05-16

**Authors:** Fan Zhang, Yaqi Yu, Hui Wang, Ying Zhang, Yan Bai, Liuyan Huang, Huachun Zhang

**Affiliations:** 1 Department of Nephrology, Longhua Hospital Shanghai University of Traditional Chinese Medicine, Shanghai, China; 2 Department of Oncology, Longhua Hospital Shanghai University of Traditional Chinese Medicine, Shanghai, China; 3 Department of Anorectology, Longhua Hospital Shanghai University of Traditional Chinese Medicine, Shanghai, China; 4 Department of Surgery, Longhua Hospital Shanghai University of Traditional Chinese Medicine, Shanghai, China; 5 Department of Cardiology, Longhua Hospital Shanghai University of Traditional Chinese Medicine, Shanghai, China; 6 Department of Nursing, Longhua Hospital Shanghai University of Traditional Chinese Medicine, 725 Wanping South Road, Xuhui District, Shanghai, China

**Keywords:** Handgrip strength, Depression, Older adults, Cross-sectional

## Abstract

**Background:**

Muscle strength and depression have been identified as independent risk factors for mortality in the elderly population. This study aimed to quantify the association between handgrip strength (HGS) and depression in community-dwelling older adults.

**Methods:**

Research data were obtained from the China Health and Retirement Longitudinal Study (CHARLS). Depression was assessed using the Center for Epidemiologic Studies Depression Scale (CESD) with a cut-off score of 20 or more. HGS was assessed with a dynamometer. Binary logistic regression and multiple linear regression models were used to test the association between HGS and depression.

**Results:**

The sample consisted of 7,036 CHARLS participants, with an average age of 68.9 ± 7.2. After adjusting for gender, age, marriage, body mass index, comorbidities, smoking, drinking, and sleep time, compared with participants in the lowest quartile of HGS, those in the second to the fourth quartile group had a 0.84- (95% CI 0.72 to 0.98), 0.70- (95% CI 0.58 to 0.84) and 0.46- (95% CI 0.35 to 0.61) fold risk of depression, respectively.

**Conclusions:**

There were a negative association between HGS and depression in community-dwelling older adults. It is critical to assess muscle strength in community older adults through accessible and valid objective measures to enhance depression screening.

**Supplementary Information:**

The online version contains supplementary material available at 10.1186/s12877-023-04034-6.

## Introduction

Depression is a common mental illness and a major cause of disability worldwide [1]. In China, the incidence of depression is on the rise year by year, with more than 95 million people suffering from depression nationwide, making it one of the "hardest hit" areas for mental health problems, mainly related to the expansion of China's aging population [2]. Studies have shown that the death and physical damage caused by suicide in depressed patients are more costly in health economics than in oncology patients and are a significant burden to individuals and society [3, 4]. In 2019, the Chinese government included depression in the "Health China (2019–2030)", with a specific action goal of slowing down the rising trend of depression by 2030 [5].

Although there is evidence of a relationship between physical activity and depression in older adults [6, 7], there is growing recognition that objective indicators of physical health may be essential. For example, several published studies have reported that objective assessments of muscle strength can help predict the onset of depression [8–10]. Handgrip strength (HGS) is a key indicator for assessing muscle strength and is associated with functional and health outcomes, including frailty [11] and mortality [12], as well as mental health [9].

Data from population-based studies assessing the relationship between HGS and depression in older adults are limited. Brooks et al. [13] used an analysis of the National Health and Nutrition Examination Survey (NHANES) dataset to show that depression was significantly associated with reduced HGS in community-dwelling adults aged 60 years and older (β = -0.19 ± 0.08) after adjusting for sociodemographic and health covariates. Furthermore, Ashdown-Franks G et al. [14] analyzed cross-sectional data from individuals aged ≥ 50 years from the World Health Organization's Study on Global Ageing and Adult Health, which included 34,129 participants, showing that weaker HGS was associated with 1.45 (95% CI: 1.12 to 1.88) times greater odds of depression. Immediately following, Zhang et al.'s cross-sectional study of 9,368 elderly Chinese inpatients found a significantly lower risk of depression as HGS increased [15]. However, the association between HGS and depression was insignificant when HGS was greater than 35.6 kg.

The association between HGS and depression based on an elderly and non-institutionalized population in the Chinese community remains unclear. Given the context of the rapid demographic transition occurring in China and the increasing number of older adults, exploring the quantitative association between HGS and depression could help identify the onset of depression early and intervene effectively. Therefore, this study aims to clarify the association between HGS and depression using data from Wave 3 (2015) of the China Health and Retirement Longitudinal Study (CHARLS). Our primary objective was to quantify the association of HGS with depression in community-dwelling older adults.

## Methods

### Study population

We used data from wave 3 of the CHARLS, which is publicly available at http://charls.pku.edu.cn. The CHARLS is a nationally representative survey involving participants aged 45 years or older and their spouses and includes an assessment of community residents' social, economic, and health status [16]. In 2015 CHARLS surveyed a total of 21,111 individuals. Of these samples, 7,036 participants aged 60 years and older were included in the current analysis after excluding participants with missing data on grip strength and depression and those aged < 60 years (Figure S1). The present study is a secondary analysis of the de-identified CHARLS public data. The Ethics Review Board approved the original CHARLS study of Peking University (IRB00001052-11,015), and all participants signed an informed consent form at the time of participation. This study followed STrengthening the Reporting of OBservational studies in Epidemiology (STROBE) reporting guidelines (see Table S1 STROBE Checklist).

### Depression assessment

The CHARLS uses the Center for Epidemiologic Studies Depression Scale (CESD) self-rating scale with ten items (Table S2). Ten items are scored on four points Likert scale (range 1 to 4), resulting in a score between 10 and 40. Scores ≥ 20 indicate the presence of depressive symptoms [17].

### Handgrip strength test

HGS was measured in kilograms by trained volunteers using a YuejianTM WL-1000 dynamometer (Nantong Yuejian Physical Measurement Instrument Co., Ltd., Nantong, China) [16]. Subjects stood and started the test using either the dominant or non-dominant hand while receiving verbal encouragement. Each subject held the ergometer at a right angle (90°) and squeezed the handle for a few seconds, taking two measurements on the right and left hand. Participants were asked to provide maximum effort to perform the measurements [18]. We used the maximum available value for measurements of the right hand.

### Covariates

The covariates were selected based on the past literature and included age, gender, marriage, body mass index (BMI), sleep duration, smoking, alcohol consumption, and comorbidities. BMI was calculated by dividing the measured weight and height in kilograms by the square of the meter and was categorized as < 18.4 kg/m^2^ (underweight), 18.5–23.9 kg/m^2^ (normal weight), and ≥ 24 kg/m^2^ (overweight) (Chinese standard) [19]. Comorbidities were aggregated based on 11 chronic physical conditions (angina, arthritis, asthma, chronic back pain, chronic lung disease, diabetes, missing teeth, hearing problems, hypertension, stroke, and visual impairment) for everyone. These conditions were based on self-report. No imputation was used to treat missing values for covariates.

### Statistical analysis

Baseline characteristics were expressed as percentages (%) of categorical variables and compared using chi-square tests. Binary logistic regression and multiple linear regression were used to calculate effect estimates and 95% confidence interval (Cl) for the association between HGS and depression. We fitted three models separately. Model 1 was unadjusted. Model 2 was adjusted for age, gender, and marriage. The model (model 3) was adjusted for gender, age, marriage, body mass index, comorbidities, smoking, drinking, and sleep time. Next, a multivariate restricted cubic spline model was used to examine the association between HGS and depression. We selected three sections at the 25th, 50th, and 75th quartiles. Finally, we performed subgroup analyses of the binary logistic regression model to test the stability of the results. Residual analysis was performed on logistic regression to test the assumptions of the regression models [20]. The variance inflation factor (VIF) was used to determine whether there was covariance between multiple variables in the logistic regression model [21]. *P* < 0.05 was considered statistically significant. SPSS 26.0 and Stata 12.0 were used for all statistical analyses.

## Results

### Baseline characteristics of study participants

Table 1 summarizes the proportion of baseline characteristics for all participants grouped according to HGS quartiles. This study included 7,036 participants (male: 47.7%, mean age: 68.9 ± 7.2 years). The mean HGS was 29.5 ± 10.0 kg. In the quartiles of HGS, we observed significant differences in all baseline covariates except age and comorbidity.

**Table 1:** Baseline characteristics of study participants according to quartiles of HGS

	Total	Q1 (≤ 22 kg)	Q2 (22, 29 kg]	Q3 (29, 36 kg]	Q4 (> 36 kg)	*P*-value
Number	N = 7,036	N = 1,765	N = 1,918	N = 1,624	N = 1,729	
Gender						< 0.001
Male	3,358 (47.7%)	243 (13.8%)	529 (27.6%)	976 (60.1%)	1,610 (93.1%)	
Female	3,678 (52.3%)	1,522 (86.2%)	1,389 (72.4%)	648 (39.9%)	119 (6.9%)	
Age group						0.702
[60, 70]	4,572 (65.0%)	1,154 (65.4%)	1,231 (64.2%)	1,048 (64.5%)	1,139 (65.9%)	
> 70	2,464 (35.0%)	611 (34.6%)	687 (35.8%)	576 (35.5%)	590 (34.1%)	
Marry						< 0.001
Married	6,227 (88.5%)	1,395 (79.0%)	1,693 (88.3%)	1,480 (91.1%)	1,659 (96.0%)	
Single	809 (11.5%)	370 (21.0%)	225 (11.7%)	144 (8.9%)	70 (4.0%)	
BMI (kg/m^2^) (*n* = 6,991)						< 0.001
≤ 18.4	351 (5.0%)	147 (8.4%)	96 (5.0%)	64 (4.0%)	44 (2.6%)	
18.5–23.9	3,263 (46.7%)	851 (48.6%)	892 (46.9%)	783 (48.5%)	737 (42.7%)	
≥ 24.0	3,377 (48.3%)	752 (43.0%)	913 (48.0%)	768 (47.6%)	944 (54.7%)	
Sleep (h) (*n* = 6,967)						< 0.001
≤ 6	3,384 (48.6%)	938 (54.2%)	933 (49.2%)	771 (43.1%)	741 (43.1%)	
> 6	3,583 (51.4%)	794 (45.8%)	965 (45.8%)	981 (56.9%)	981 (56.9%)	
Comorbidities						0.223
None	6,933 (98.5%)	1,745 (98.9%)	1,892 (98.6%)	1,592 (98.0%)	1,704 (98.6%)	
At least one	103 (1.5%)	20 (1.1%)	26 (1.4%)	32 (2.0%)	25 (1.4%)	
Smoking (*n* = 4,545)						< 0.001
No	3,968 (87.3%)	1395 (96.6%)	1383 (95.8%)	783 (84.7%)	407 (55.4%)	
Yes	577 (12.7%)	49 (3.4%)	60 (4.2%)	141 (15.3%)	327 (44.6%)	
Drinking (*n* = 7,032)						< 0.001
No	4,501 (64.0%)	1,448 (82.1%)	1,436 (74.9%)	957 (59.0%)	660 (38.2%)	
Yes	2,531 (36.0%)	315 (17.9%)	481 (25.1%)	666 (41.0%)	1,069 (61.8%)	

*HGS* handgrip strength, *Q* quartile, *BMI* body mass index

### Relationship between HGS and depression

**Fig. 1:**
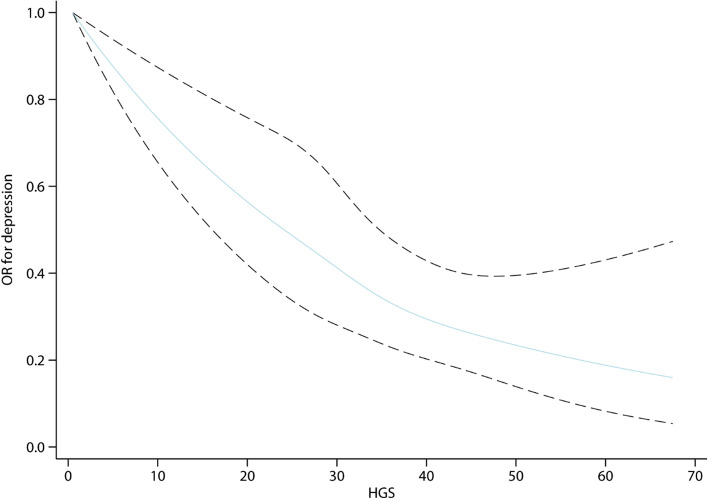
Adjusted restricted cubic spline model of the association between HGS and depression

Table S3 shows the results of the univariate logistic regression analysis. After adjusting for gender, age, marry, body mass index, comorbidities, smoking, drinking, and sleep time, every SD (10.0) increase in HGS was associated with a 26% lower risk of developing depression (OR: 0.74, 95% CI: 0.67 to 0.81). When comparing with the lowest quartile of HGS, the multivariate ORs for occurrence of depression were 0.84 (95% CI: 0.72 to 0.98) for Q2, 0.70 (95% CI: 0.58 to 0.84) for Q3, 0.46 (95% CI: 0.35 to 0.61) for Q4 (*P*_for trend_ < 0.001) (Table 2). Restricted cubic spline curves approximating a "J" shape show a negative association between HGS and depression risk (Fig. 1).

**Table 2:** Odds ratio for HGS and depression

	N	Model 1	Model 2	Model 3
Per SD (10.0 kg) increase		0.64 (0.61, 0.68)	0.67 (0.63, 0.72)	0.74 (0.67, 0.81)
Per 1-unit increase		0.96 (0.95, 0.96)	0.96 (0.96, 0.97)	0.97 (0.96, 0.98)
Quartiles				
Q1 (≤ 22 kg)	1,765	Ref	Ref	Ref
Q2 (22, 29 kg]	1,918	0.76 (0.66, 0.86)	0.77 (0.68, 0.89)	0.84 (0.72, 0.98)
Q3 (29, 36 kg]	1,624	0.60 (0.52, 0.69)	0.65 (0.55, 0.75)	0.70 (0.58, 0.84)
Q4 (> 36 kg)	1,729	0.32 (0.27, 0.37)	0.36 (0.30, 0.44)	0.46 (0.35, 0.61)
*P*_for trend_		< 0.001	< 0.001	< 0.001

Data are presented as odds ratio (95% confidence interval). *SD* standard deviation, *Q* quartiles, *Ref*. reference

Model 1 unadjusted

Model 2 adjusted for age, gender, and marriage

Model 3 adjusted for gender, age, marriage, body mass index, comorbidities, smoking, drinking, and sleep time

We performed subgroups analyses to stratify the association between HGS and depression by gender, age, marriage, BMI, drinking, sleep time, and comorbidities. No interaction was found between subgroup variables and the association between HGS and depression, except gender (Table 3). The difference in the association between HGS and depression by gender was statistically significant (*P*_for interaction_: 0.034). Given this result, we calculated the probability of handgrip strength predicting depression stratified by gender (male: 79.2%; female: 61.8%, Table 3). In addition, we dichotomized participants' HGS (HGS defined as < 28 kg for men and < 18 kg for women [22]) as recommended by the Asian sarcopenia working group, which, after adjusting for variables, produced similar results that low HGS was associated with an elevated risk of depression in older adults (Table S4 and Figure S2).

**Table 3:** Multivariable-adjusted odds ratio for the association between quartiles of HGS and depression by subgroups

	N	Q1	Q2	Q3	Q4	*P*_for trend_	*P*_for interaction_
Total	7036	Ref	0.84 (0.72, 0.98)	0.70 (0.58, 0.84)	0.46 (0.35, 0.61)	< 0.001	—
Gender^a^							0.034
Male	3358 (47.7%)	Ref	0.62 (0.34, 1.15)	0.49 (0.28, 0.86)	0.32 (0.19, 0.55)	< 0.001	
Female	3678 (52.3%)	Ref	0.85 (0.73, 1.00)	0.72 (0.58, 0.89)	0.56 (0.36, 0.88)	< 0.001	
Age (y)							0.372
[60, 70]	4572 (65.0%)	Ref	0.81 (0.67, 0.98)	0.71 (0.56, 0.90)	0.43 (0.30, 0.61)	< 0.001	
> 70	2464 (35.0%)	Ref	0.91 (0.70, 1.19)	0.68 (0.49, 0.94)	0.52 (0.33, 0.83)	0.003	
Marriage							0.718
Married	6227 (88.5%)	Ref	0.83 (0.70, 0.99)	0.69 (0.56, 0.85)	0.47 (0.35, 0.63)	< 0.001	
Single	809 (11.5%)	Ref	0.86 (0.58, 1.29)	0.75 (0.40, 1.42)	0.33 (0.11, 1.02)	0.076	
BMI (kg/m^2^)							0.625
≤ 18.4	351 (5.0%)	Ref	0.63 (0.29, 1.38)	0.36 (0.10, 1.30)	0.35 (0.07, 1.79)	0.074	
18.5–23.9	3263 (46.7%)	Ref	0.87 (0.69, 1.09)	0.72 (0.53, 0.97)	0.49 (0.32, 0.76)	0.001	
≥ 24.0	3377 (48.3%)	Ref	0.83 (0.67, 1.04)	0.70 (0.54, 0.90)	0.45 (0.30, 0.65)	< 0.001	
Sleep (h)							0.686
≤ 6	3384 (48.6%)	Ref	0.88 (0.71, 1.09)	0.71 (0.55, 0.93)	0.54 (0.37, 0.79)	0.001	
> 6	3583 (51.4%)	Ref	0.79 (0.62, 1.00)	0.68 (0.51, 0.91)	0.39 (0.26, 0.59)	< 0.001	
Comorbidities							0.833
None	6933 (98.5%)	Ref	0.83 (0.71, 0.97)	0.69 (0.57, 0.84)	0.46 (0.35, 0.61)	< 0.001	
At least one	103 (1.5%)	Ref	2.11 (0.37, 12.21)	1.08 (0.11, 10.44)	0.44 (0.03, 6.34)	0.608	

Data are presented as odds ratio (95% confidence interval); *Q *quartiles, *Ref. *reference

Models were adjusted for gender, age, marriage, body mass index, comorbidities, smoking, drinking, and sleep time except the subgroup variable itself

^a^Given the significant interaction of gender, we additionally calculated predicted probabilities, male is 79.2%, and female is 61.8%

According to the VIF, there was no evidence of multicollinearity in any of the models analyzed by logistic regression (VIF < 4). Visual inspection of the residual plots with fitted values indicated that the linearity assumptions of the regression models were met.

## Discussion

From the national data of CHARLS, we found that after controlling for potential confounders, older adults with the highest HGS had a 54% lower risk of depression than those with the lowest HGS (OR: 0.46; 95% CI: 0.35 to 0.61). Further exploratory investigations showed that this association was independent of age, marriage, BMI, sleep duration, and comorbidity, but there may be an interaction with gender, which allows the association between HGS and depression in older adults to be established.

### Comparison with other studies

Similar to a recent cross-sectional study on hospitalized older adults in China, there was a non-linear relationship between HGS and depression in community-dwelling older adults [15]. Another survey of middle-aged and older adults in 18 European countries showed that men and women in the second, third, and fourth quartiles of HGS were less likely to have depressive symptoms than men and women in the first quartile of HGS. This is consistent with our study. The difference is that the study by Marques A et al. [23] used the HGS thresholds delineated by the European Working Group on Sarcopenia in Older People (i.e., HGS < 30 kg in men and < 20 kg in women), whereas our study was based on the Asian Working Group on Muscular Dystrophy. The low HGS cut-offs across geographic regions still yielded similar results, reinforcing the negative association between handgrip strength and depression. In addition, Marques A et al. [24] conducted a pooling of evidence that included the results of a systematic review of 21 studies suggesting a positive effect of muscle strength on reducing depression, and a meta-analysis also reported an inverse association between muscle strength and depression (OR: 0.85; 95% CI: 0.80 to 0.89). Moreover, in a large cohort study, Kandola AA et al. [25] with a 7-year follow-up of 152,978 United Kingdom Biobank participants, Low and medium HGS was associated with 1.410 (95% CI: 1.335 to 1.490) and 1.126 (95% CI: 1.066 to 1.189) higher odds of the common mental disorder compared to high HGS.

Interestingly, an interaction between gender and HGS and depression was found in the exploratory analysis of this study. The potential reason may be that sex hormone levels (e.g., testosterone and estrogen) play a crucial role in muscle development, and differences in estrogen concentrations between the sexes may explain this interaction effect. In addition, regression analysis showed that HGS had a predictive probability of 61.8% for developing depression in women, which was lower than in older men (79.2%). It has been shown that women have a more prominent decline in muscle strength than men after age 55 [26], possibly due to decreased muscle protein synthesis post-menopause [27], and that weak muscle strength is associated with poor mental health, including an increase in depressive symptoms [28].

### Possible explanations and implications

The negative association of HGS with depression can be explained by the fact that, on the one hand, low HGS leads to a limitation of motor function in individuals, as well as to physical weakness and reduced physical activity, inducing inflammation, especially in older age groups [29, 30]. The inflammatory factor theory suggests that the inflammatory response in the body occurs with the production of large amounts of cytokines that promote the inflammatory response, such as interleukins and tumor apoptotic factors, which enter the brain and affect neurotransmitters, neuromodulators, or neural circuits related to mood-emotion regulation, thus producing the symptoms of depression [31, 32]. On the other hand, HGS is a key indicator for assessing sarcopenia, a risk factor for increased depression [33].

With this in mind, the current study has several clinical implications. Since HGS predicts the onset of depression, routine screening for HGS should be incorporated into the prevention and management process of depression. More importantly, HGS can be reversed or maintained by exercise training. Meta-analyses based on randomized control trials have shown that aerobic and resistance training is beneficial in improving HGS [34–37]. Several cohort studies have also demonstrated the benefits of physical activity for depression [38–40]. Therefore, promoting physical activity in people at high risk for depression is a public health issue that needs appropriate attention and intervention.

### Limitations

First, because it is a cross-sectional study, this study cannot infer causality. Second, given that the CESD is a self-report questionnaire, the diagnosis of depression may not be very accurate. Third, although we considered some of the confounding factors associated with depression, we did not consider inflammatory factors included, which are important indicators of the occurrence of depression. However, the negative association of HGS with depression remained in the study by Wu et al. after correction for inflammatory markers (C-reactive protein) [41]. Fourth, due to the high number of missing HGS retest data from CHARLS, we included only the first measurement, which may have overestimated the association between HGS and depression.

## Conclusion

This cross-sectional CHARLS-based study suggests that low HGS is associated with an increased risk of depression in community-dwelling older adults. The results of this study emphasize that future research and clinical practice should apply available, accessible, and valid objective measures to assess muscle strength to enhance depression screening, complemented by targeted measures to reduce the risk of depression.

## Supplementary Information

**Additional file 1: Figure S1.** Flow chart of study participants. **Figure S2.** Adjusted restricted cubic spline model of the association between HGS and depression. **Table S1.** STROBE Statement—checklist of items that should be included in reports of observational studies. **Table S2.** The item of the Center for Epidemiological Survey Depression Scale. **Table S3.** Comparison of baseline characteristics of study participants. **Table S4.** Multivariable-adjusted odds ratio for the association between HGS and depression by gender.

## Data Availability

The datasets generated during and/or analyzed during the current study are available in the CHARLS repository, http://charls.pku.edu.cn.

## Abbreviations

HGS: Handgrip strength
CHARLS: China Health and Retirement Longitudinal Study
CESD: Center for Epidemiologic Studies Depression Scale
NHANES: National Health and Nutrition Examination Survey
STROBE: STrengthening the Reporting of OBservational studies in Epidemiology
BMI: Body mass index
VIF: Variance inflation factor
95% Cl: 95% Confidence interval
